# Self-Adaptive Strategy Based on Fuzzy Control Systems for Improving Performance in Wireless Sensors Networks

**DOI:** 10.3390/s150924125

**Published:** 2015-09-18

**Authors:** Vicente Hernández Díaz, José-Fernán Martínez, Néstor Lucas Martínez, Raúl M. del Toro

**Affiliations:** 1 Centro de Investigación en Tecnologías Software y Sistemas Multimedia para la Sostenibilidad (CITSEM), Universidad Politécnica de Madrid, Calle Alan Turing 3, 28031 Madrid, Spain; E-Mails: jf.martinez@upm.es (J.-F.M.); nestor.lucas@upm.es (N.L.M.); 2 Centro de Automática y Robótica, Universidad Politécnica de Madrid, Carretera Campo Real Km. 0.2, 28500 Arganda del Rey, Spain; E-Mail: raul.deltoro@car.upm-csic.es

**Keywords:** wireless sensors networks, fuzzy logic, self-adaptive, cyber-physical systems

## Abstract

The solutions to cope with new challenges that societies have to face nowadays involve providing smarter daily systems. To achieve this, technology has to evolve and leverage physical systems automatic interactions, with less human intervention. Technological paradigms like Internet of Things (IoT) and Cyber-Physical Systems (CPS) are providing reference models, architectures, approaches and tools that are to support cross-domain solutions. Thus, CPS based solutions will be applied in different application domains like e-Health, Smart Grid, Smart Transportation and so on, to assure the expected response from a complex system that relies on the smooth interaction and cooperation of diverse networked physical systems. The Wireless Sensors Networks (WSN) are a well-known wireless technology that are part of large CPS. The WSN aims at monitoring a physical system, object, (e.g., the environmental condition of a cargo container), and relaying data to the targeted processing element. The WSN communication reliability, as well as a restrained energy consumption, are expected features in a WSN. This paper shows the results obtained in a real WSN deployment, based on SunSPOT nodes, which carries out a fuzzy based control strategy to improve energy consumption while keeping communication reliability and computational resources usage among boundaries.

## 1. Introduction

Cyber-Physical Systems (CPS) aim at developing cross-domain solutions from the interaction of the physical world, the world made of real daily objects like cars, buildings, robots, medical wearable devices, traffic lights and so on, and the virtual world, the world where computational representations of such objects exist. CPS will enable the readily development of applications for smart transportation, e-Health, smart grid and factories of the future domains, among others, by orchestrating, managing and controlling the physical objects and their emergent behavior by means of their virtual computational representation.

In recent years, advances in wireless communication technologies and their benefits [1], have entailed a greater application of these technologies for supporting communication in CPS. Thus, the Wireless Sensors Networks (WSN), a well-known wireless technology that connects sensors and actuators, are becoming part of large CPS. The WSN aims at monitoring a physical system and relaying data to a targeted processing element. However, as mentioned in [2] and considering the CPS overall design requirements in [3] and the time requirements to be fulfilled when interacting with physical objects [4], there are relevant challenges that WSN have to overcome in order to be fully integrable in CPS, and the energy consumption and the communication reliability are two important aspects targeted in this paper.

Reliability of wireless communications can be affected by several factors such as the environment, the frequency and modulation used, and even the orientation of the sensor antenna introduces radio irregularities [5]. In the current available literature is possible to find several studies that analyze the level of influence of some of these factors in wireless communication, also suggesting methods and techniques to address them and thus improve the reliability and robustness of wireless communication technologies.

Kotian *et al*. [6] have been working on the problem of Transmission Power Control (TPC) for WSN by measuring the Received Signal Strength Indicator (RSSI) in order to analyze the link quality between the nodes and comparing different filtering techniques for link quality prediction. Mahmood *et al*. [7] have studied the reliability of protocols used in WSN concluding among other things that cross layer design should be further exploited to achieve reliability. Kusy *et al*. [8] proposed a dual radio network architecture to improve communication reliability in WSN, with a minor increase in energy consumption.

The research and development carried out in this work are based on the hypothesis that the adaptive connectivity between wireless nodes in the network and a certain number of neighboring nodes, improves reliability of the network communications. The main scientific and technical contributions are the use of intelligent control techniques, such as fuzzy control, for developing the adaptation strategy and its validation in a physical scenario.

The theoretical foundations for the proposed contributions has been introduced in a previous work [9]. Briefly, the likelihood for a node in a WSN to reach the WSN sink, which collects and processes the nodes data, depends on the number of neighbors. A higher number of neighbors implies a higher likelihood. In [9] you can find the rationale for an optimal number of neighbors that guarantees an acceptable likelihood. The optimal number of neighbors depends on the WSN deployment area and the density of nodes (number of nodes per m2). Therefore, each WSN node should adapt its power transmission to guarantee the WSN optimal number of neighbors, thus achieving a better energy saving. The current work goes an step forward by presenting the results under the basement of empirical evidences and by also applying engineering methodologies for the design and development process.

The organization of this work are presented as follows: Section 2 presents the system design, Section 3 describes the experiments and the equipment that has been used, Section 4 analizes the obtained results and finally, some conclusions about the work results are shown in Section 5.

## 2. System Design

The system presented in this section is a WSN comprised of several SunSPOT [10] nodes, a product manufactured by Oracle. It is an outcome from the authors participation in the European research project “Design, Monitoring, and Operation of Adaptive Networked Embedded Systems” (DEMANES). It aims at improving the overall energy consumption of the network while keeping communication reliability and computational resources usage among acceptable boundaries. As mentioned previously, communication reliability and energy consumption are some of the WSN key aspects that could prevent it from being suitable for CPS based applications.

**Figure 1:**
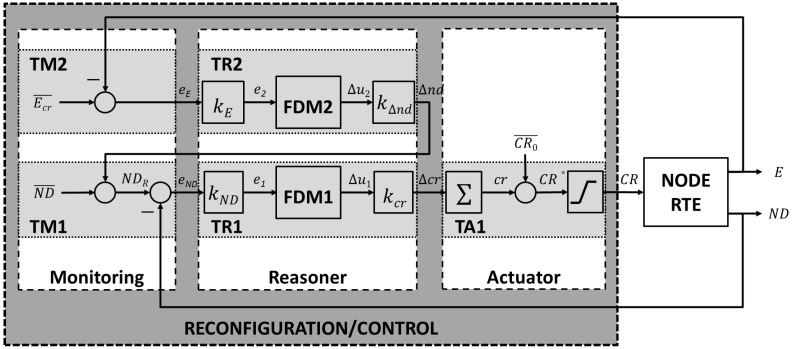
Control system design for self-adapting the Wireless Sensors Networks (WSN) nodes transmission power considering the number of neighbors and the battery level.

The software modules deployed in the SunSPOT nodes are based on the control system design that is depicted in Figure 1 and fully described in [9]. It accomplishes a self-adaptive system through two feedback control loops as suggested by Yuriy Brun *et al*., in [11].

**Figure 2:**
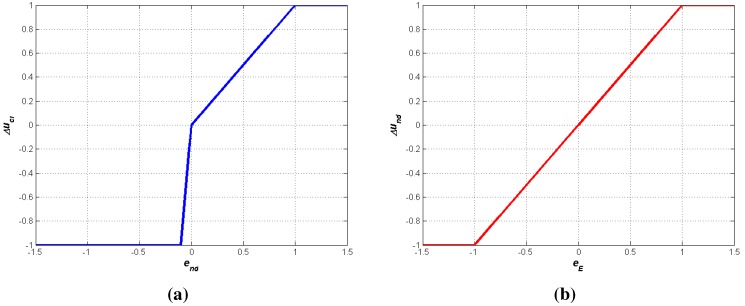
Fuzzy transfer functions. (**a**) FDM1; (**b**) FDM2.

A primary feedback control loop consists of tasks *TM1*, *TR1* and *TA1* in Figure 1 and manages the node transmission power considering both its real and targeted number of neighbors. A secondary feedback control loop consists of tasks *TM2* and *TR2* in Figure 1 and manages the node targeted number of neighbors considering the battery level. The objectives of each task are:

- *TM1* is run periodically at a frequency discussed in the following sections. It calculates the node targeted number of neighbors $ND_{R}$ by adding the WSN optimal value $\bar{ND}$ and the change to be applied $\Delta_{nd}$ estimated by *TR2*, based on the battery level. Then samples the real number of neighbors *ND* using the node run-time environment, calculates the difference ($e_{ND}$) between the real and the targeted number of neighbors and decides if the *TR1* must be triggered.
- *TR1* decides the change to be applied on the node transmission power so that the real and the targeted number of neighbors of the node become equals or quite similar. *TR1* decision is driven by *FDM1*. *FDM1* is a decision making function based on fuzzy logic, depicted in Figure 2b and briefly explained below. Finally *TR1* is executed when *TM1* considers appropriate.
- *TA1* effectively establishes the node transmission power based on the *TR1* output. It is always run once *TR1* finishes. $\bar{CR_{0}}$ in Figure 1 is the initial value for the node transmission power. The *TR1* consecutive outputs are increments or decrements that *TA1* adds up to get the desired transmission power as a deviation from $\bar{CR_{0}}$. The last building block in *TA1* guarantees that the transmission power will be a value supported by the node run-time environment.
- *TM2* is run periodically at a frequency discussed in the following sections. It samples the node real battery level *E* using the node run-time environment, computes $e_{E}$ as the difference between the critical battery level $\bar{E_{cr}}$ and the real battery level and triggers *TR2*.
- *TR2* decides the change ($\Delta_{nd}$) to be applied to the WSN optimal number of neighbors $\bar{ND}$, thus influencing *TM1*. The decision making process is driven by *FDM2*, a decision making function based on fuzzy logic depicted in Figure 2b and briefly explained below.

*FDM1* and *FDM2* functions represent the input-output relationship of single input-single output decision-making functions based on fuzzy logic. To model a WSN and the appropriate response to any possible failure or change in the WSN environment is overwhelming, and fuzzy-based solutions are likely to provide optimal and feasible achievements. In [12], several algorithms for controlling a WSN topology are compared and those based on fuzzy-logic show a better trade-off between feasibility and communication reliability. The design of the functions were introduced in [9]. The input and output ranges for both function are normalized between −1 and 1. The functions were designed based on engineering expertise and criterion, and after observation of the system behavior in a simulated environment.

*FDM1* determines the increment on the transmission power to be applied depending on the difference between the desired number of neighbors and the present one. If the present number of neighbors is greater than the desired one, the transmission power will be increased, and decreased otherwise. For a negative input the function has larger slope than for positive values. The idea is to get large changes for negative values (the present number of neighbors is greater than the desired one $ND_{R}$) in order to save energy consumption due to lower transmission power required. By other side, *FDM2* adjusts the desired number of neighbors for each WSN node and is linear in the range between −1 and 1. The desired number of neighbors for a node will be decreased if the battery level drops below a critical value.

**Figure 3:**
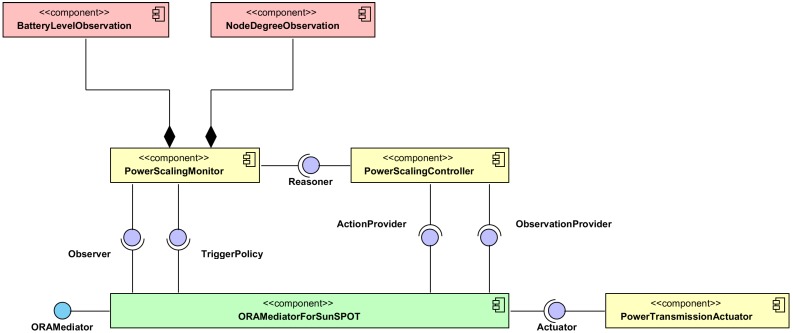
Software components accomplishing the self-adaptive system improving the energy consumption while keeping the communication connectivity.

In addition, the Figure 1 also provides a different view of the system as consisting of three blocks: ***Monitoring*** (comprises the tasks *TM1* and *TM2*), ***Reasoner*** (comprises the tasks *TR1* and *TR2*) and ***Actuator*** (comprises the task *TA1*), which complies with the architectural model MAPE-K (Monitor, AnalyZe, Plan, Execute, Knowledge) [13] and has driven the software design of the system. The Figure 3 proposes a general software design for accomplishing self-adaptive systems based on MAPE-K. It is based on the one fully described in [14], customized for the SunSPOT platform. The components ***PowerScalingMonitor***, ***BatteryLevelObservation*** and ***NodeDegreeObservation*** in Figure 3 shape the ***Monitoring*** block in Figure 1. The component ***PowerScalingMonitor*** monitors, at a specific rate, the number of neighbors (also called *node degree*) and the battery level of a node, and also decides if the change in any of those values is significant enough to run the reasoner, the component based on fuzzy control that establishes the desired transmission power. The components ***BatteryLevelObservation*** and ***NodeDegreeObservation*** measure respectively the battery level and the number of neighbors of a node. The component ***PowerScalingController*** accomplishes the ***Reasoner*** block in Figure 1. This component’s parameters must be tuned whenever a change in the dynamics of the control system is needed. The component ***PowerTransmissionActuator*** carries out the ***Actuator*** block in Figure 1, is able to modify the transmission power of a node based on the desired value specified by the invoking component. Finally, the component ***ORAMediatorForSunSpot*** enables interaction among the rest of components. It behaves as a broker decoupling observers, reasoners and actuators.

**Figure 4:**
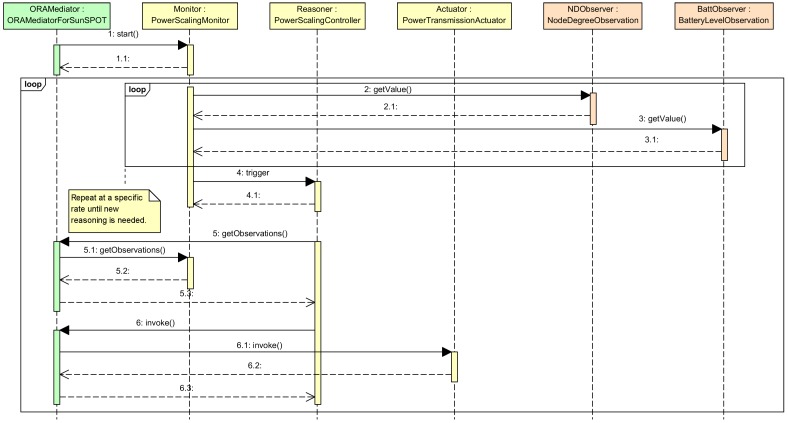
UML sequence diagram depicting basic components interaction.

Whenever a component is registered in the ***ORAMediatorForSunSpot***, the rest of registered components are aware of that, thus they can access the new one if needed. The new components are registered by means of the interface *ORAMediator*. The regular interactions among components is depicted in the UML sequence diagram in Figure 4. The ***ORAMediatorForSunSpot*** will start the ***PowerScalingMonitor*** component by means of the *TriggerPolicy* interface. That component will measure periodically the number of neighbors and the battery level of a node and will decide, based on such values, if it is worth triggering the reasoner to make new decisions, by means of the *Reasoner* interface. The ***PowerScalingController*** component will request the latest measures by means of the *ObservationProvider* interface whenever triggered. The ***ORAMediatorForSunSpot*** component captures the request, gets the data from the observer by means of the *Observer* interface and finally fulfils the ***PowerScalingController*** request. Then, the ***PowerScalingController*** will run the algorithm depicted in the block *Reasoner* in Figure 1, including *FDM1* and *FDM2* outlined in Figure 2a,b, and could request a change in the node transmission power by means of the *ActionProvider* interface whenever suitable. The ***ORAMediatorForSunSpot*** component captures the request and invokes the proper actuator, the ***PowerTransmissionActuator*** in this case, to fulfill the request.

The components in the Figure 3 have been developed in Java language for the SunSPOT platform, a Java Micro Edition virtual machine called *squawk*. The commercially available WSN products are very diverse. There are products with low memory capacity and microcontroller speed, like G-Node [15] or Waspmote [16], an Arduino based platforms. But there are also more powerful hardware platforms like Intel Galileo Gen 2 [17] or Raspberri PI [18], that were not at first designed for becoming a WSN node but they can be very suitable whenever a WSN node has to run complex algorithms for making decisions, data aggregation and so on. The SunSPOT capabilities are better than those from resource constrained solutions like G-Node, but worse than those offered by powerful platforms. The SunSPOT platform was selected because it is Java programmed, making easier the development phase of a solution, and it is a well known platform for the authors.

The control strategy that have been carried out and described in this paper aims at consuming the less resources as possible and minimizing the network overhead. On the one hand, the reasoning algorithms do not require either complex and overloading processing instructions, and are presented in the Figure 2a,b. The observed parameters that drive the control strategy, the battery level and the number of neighbors, are neither high resource consuming elements. The former is directly obtained by the tools provided by the SunSPOT platform. The latter could be estimated considering the information provided by the node routing protocol or the messages going across the node, however, a discovery protocol was carried out as the accuracy of the number of neighbors provided by the SunSPOT platform was not good enough.

## 3. Experimental Set-up

We have defined a set of experiments to test the improvements, if any, achieved by using the proposed fuzzy control based self-adaptive system in a WSN. There are four main parameters that rule the behavior of the self-adaptive system deployed in the SunSPOT nodes: $ND_{R}$, $\xi_{ND}$, $k_{CR}$ and $E_{CR}$, which have been modified in the different experiments to observe each parameter impact on the system efficiency.

- $ND_{R}$ is the number of neighbors, a reference value, that the node must have. That value could change over time and decrease if a node battery level drops below the critical value $E_{CR}$. The initial value for $ND_{R}$ depends on the number of nodes in the WSN and the deployment area, as mentioned in previous sections.
- $\xi_{ND}$ determines when the difference ($e_{ND}$) between a node $ND_{R}$ and its real number of neighbors is significant enough to trigger the reasoner, avoiding useless executions of task *TR1*. More precisely, the task *TM1* in the component ***PowerScalingMonitor*** will trigger the task *TR1* in the component ***PowerScalingReasoner*** when $|e_{ND}\left(k\right)|-\xi_{ND}>0$, where $|e_{ND}\left(k\right)|$ represents the absolute value of the error $e_{ND}$ in the *k* instant.
- $k_{CR}$ amplifies *FDM1* output, the required change in the node transmission power to meet $ND_{R}$. This parameter influences how long it takes the system to get $ND_{R}$ number of neighbors. For instance, a high value for $k_{CR}$ in a system where the number of neighbors of a node changes significantly, e.g., due to environmental changes, implies that it will take less system cycles to recover $ND_{R}$.
- $E_{CR}$ is a system reference value that establishes the battery level value that suggests a higher energy consumption saving to extend the battery lifetime. When the real battery level drops below $E_{CR}$, the system should reduce energy consumption by reducing the transmission power, acting on the reference value for the number of neighbors of the node.

In this paper we focus on the first three ones. We also did two control experiments with the self-adaptive system inactive to compare the system performance when the self-adaptive system is on and off. The total number of experiments was ten: two with a fixed transmission power, and eight with an adaptive transmission power ruled by the proposed control system and a set of different parameters to test their performance. The parameters used for these control experiments are shown in Table 1.

**Table 1:** Parameter values used in the experiments.

Experiment	$ND_{R}$	$\xi_{ND}$	$k_{CR}$	$E_{CR}$	$P_{TX}$
e01	—	—	—	—	−3 dBm
e02	—	—	—	—	−15 dBm
e03	2	0	1	150	—
e04	2	0	3	150	—
e05	2	1	3	150	—
e06	3	1	3	150	—
e07	3	0	3	150	—
e08	3	0	1	150	—
e09	3	1	1	150	—
e10	2	1	1	150	—

**Figure 5:**
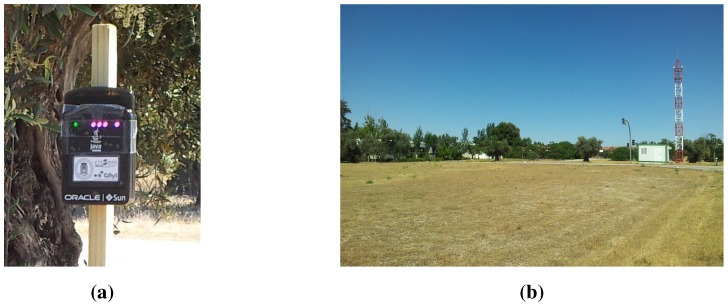
Experiment deployment; (**a**) One of the sensor nodes used in the experiments; (**b**) Deployment area.

The nodes used in the experiments were eight SunSPOT wireless sensors nodes [10], like the one in Figure 5a. All the SunSPOT nodes used in the experiments use the same hardware revision, that is revision 8.2 for the main board, and revision 8.1 for the sensor board. They were updated to the developer version codenamed “*teal-120517*” in order to solve an issue with the readings of the battery values in the previous SunSPOT SDK release.

We also used a SunSPOT base station, which is just like any other SunSPOT device, but without the sensor board. The SunSPOT base station was attached to a computer through the USB port, and was used as a sink for the status messages sent by the sensor nodes, as well as to enable the remote management of the control system parameters used in the deployed sensor nodes. It also took a passive role in the neighbor discovery protocol, and an active role in the routing protocol. The status messages had a fixed length of 60 bytes, and carried out information regarding the round of the experiment, the time-stamp, the current number of neighbors, the maximum and available battery capacity, the configuration parameters used in the associated round and the next hop as indicated by the node routing protocol.

All the SunSPOTs are equipped with a CC2420 transceiver that provides radio capabilities using the 2.4 GHz for the IEEE 802.15.4. This transceiver allows a transmission power range from −3 dBm to −32 dBm when using channel 26, and from 0 dBm to −32 dBm for the rest. The range is discrete, and the available transmission powers are shown in Table 2. For the experiments we have used channel 26, so in our case the maximum transmission power is limited to −3 dBm.

**Table 2:** Available transmission powers in the SunSPOT nodes.

Channel	Available Transmission Powers (dBm)
11 to 25	−32, −31, −30, −25, −22, −19, −17, −15, −13, −12, −11, −10, −9, −8, −7, −6, −5, −4, −3, −2, −1, 0
26 (default channel)	−32, −31, −30, −25, −22, −19, −17, −15, −13, −12, −11, −10, −9, −8, −7, −6, −5, −4, −3

**Figure 6:**
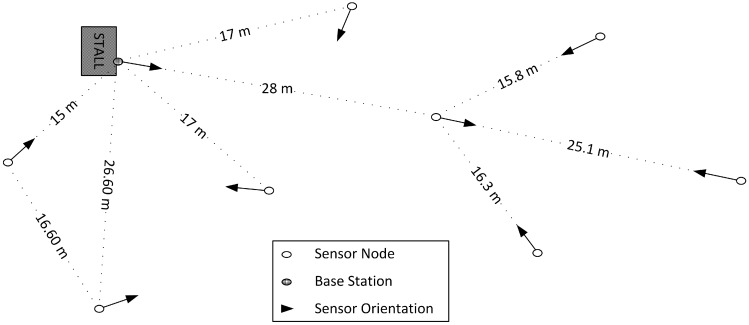
Experiment deployment.

The nodes were deployed in an outdoor area at the facilities of the Centro de Automática y Robótica in Arganda del Rey, as shown in Figure 5a,b. The distribution and heights of the nodes were mostly random, trying to force the use of the multi-hop routing capabilities provided by the SunSPOTs, so we could expect the creation of groups of nodes. A scheme of the deployment is shown in Figure 6. The heights were above one meter in all the nodes but in one, which was closer to the ground, at a height of about 40 cm. The base station was fixed to the outside of the stall, at an approximate height of 1.5 m.

Figure 6 also shows the orientation of the sensors, and therefore of their inverted-F antennas. As explained in [5], the orientation of the antennas, along with the irregularity in their radiation patterns introduce a non-negligible phenomenon that has an impact in the strength of the received signal (RSSI). The orientations were also selected randomly.

In the previous section we have introduced the idea that the control system is executed periodically. The interval used in the experiments was fixed to 20 s in order to guarantee the neighbor discovery and the route establishment for all the status messages generated in each iteration. A shorter interval may be used taking into account the number of messages that can be generated in each iteration and the values obtained in [19]. Moreover, each sensor node was configured to sent a status message addressed to the base station at the end of each iteration of the control system.

## 4. Results

The purpose of the experiments was to test the performance of the fuzzy control based self-adaptive system regarding the energy consumption and the communications reliability. The energy consumption is directly related to the battery discharge rate, and the communications reliability is measured by the error rate. In the following subsections we will discuss the results for both.

### 4.1. Energy Consumption

**Figure 7:**
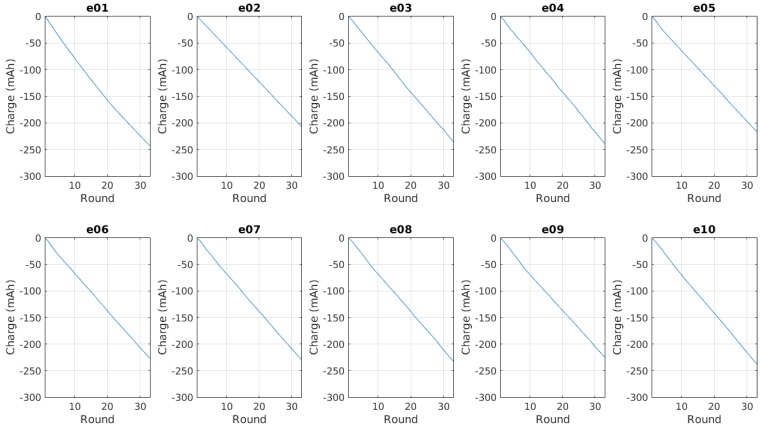
Evolution of the total charge per experiment, relative to the first value of each one.

Figure 7 shows the evolution of the available battery capacity for the whole network. The battery capacity for each node is added to the total for each iteration of the control system, and them the resulting value is calculated in relation to the first aggregated value for each experiment. The graphs have been restricted to the shortest experiment, so we can easily compare the results from one experiment to the others.

We can measure the performance of the system by performing the numerical integration of the curve obtained in each experiment, as expressed in Equation (1). The network consumes less energy when $J_{e}$ is lower.

(1) $$J_{e}=\sum \left|C_{e}\right|$$

A first look at the graphs also shows a linear trend like the one expressed in Equation (2).

(2) $$C_{e}=\alpha_{e}-\beta_{e}x$$

**Figure 8:**
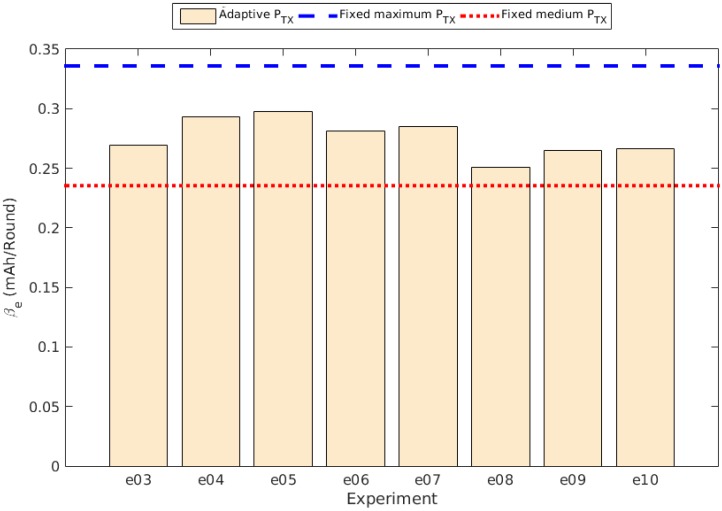
Slope ($\beta_{e}$) comparison between experiments.

**Figure 9:**
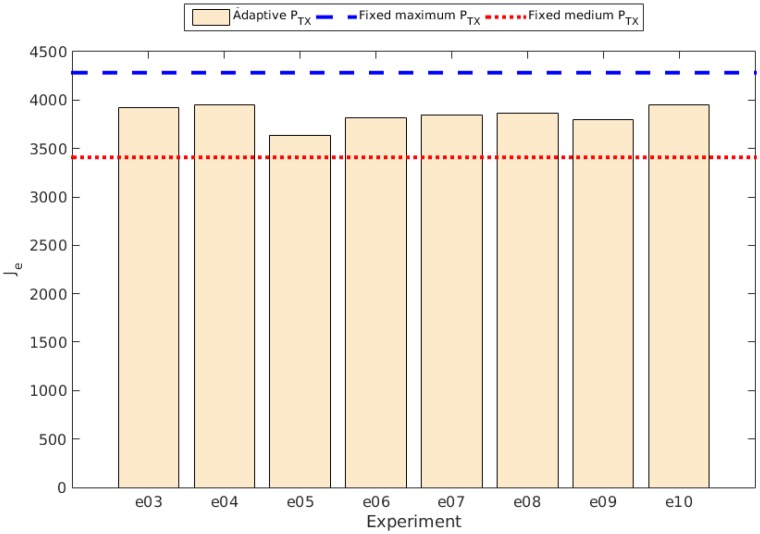
$J_{e}$ comparison between experiments.

The slope determined by $\beta_{e}$ is obviously related to the discharge rate, as it represents the direct proportionality between the charge and the time, in this case expressed as the round, or the iteration of the control system. Figure 8 shows the different values of $\beta_{e}$ obtained in the experiments with the control system running compared to the values obtained in the experiments with fixed transmission power ($P_{TX}$), while Figure 9 shows the comparison of the $J_{e}$ values. The values for $\beta_{e}$ and $J_{e}$ are shown in Table 3.

**Table 3:** Battery consumption slope and discrete integration per experiment.

Experiment	$\beta_{e}$	$J_{e}$	$\frac{\beta_{e}}{\max_{\beta_{e}}}\left(\%\right)$	$\frac{J_{e}}{\max J_{e}}\left(\%\right)$
e01	0.3358	4283.19	100.00	100.00
e02	0.2353	3408.00	70.07	79.57
e03	0.2691	3921.53	80.14	91.56
e04	0.2928	3947.76	87.19	92.17
e05	0.2976	3639.89	88.62	84.98
e06	0.2815	3817.46	83.83	89.13
e07	0.2848	3846.54	84.81	89.81
e08	0.2511	3865.22	74.48	90.24
e09	0.2647	3798.83	78.83	88.69
e10	0.2665	3952.63	79.36	92.28

The results show an improvement in the energy consumption relative to the experiment with a fixed transmission power set to the available maximum value. The percentage changes relative to $J_{e}$ and $\beta_{e}$ are also shown in Table 3. As can be observed, although there is a clear improvement with respect to the use of a fixed maximum transmission power, all the adaptive experiments resulted in percentage changes lower than the one using a fixed transmission power set to a medium value.

### 4.2. Communication Reliability

**Figure 10:**
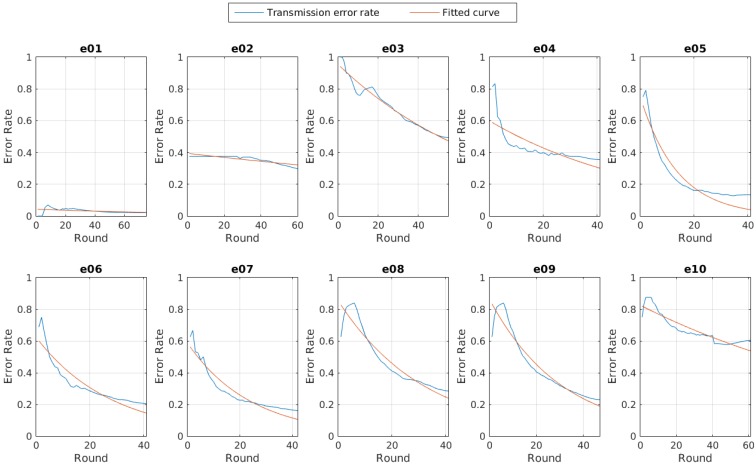
Evolution of the total transmission error rate per experiment.

In Figure 10 we can see the evolution of the error rate per experiment, and the fitted curve to each one.

The curve for the experiments where the self-adaptive system is running seems to follow a negative exponential equation like the one described in Equation (3).

(3) $$\epsilon_{c}=\alpha_{c}e^{-\beta_{c}}$$

We estimate the performance regarding the error rate using the numerical integration as defined in Equation (4).

(4) $$J_{c}=\sum \epsilon_{c}$$

**Figure 11:**
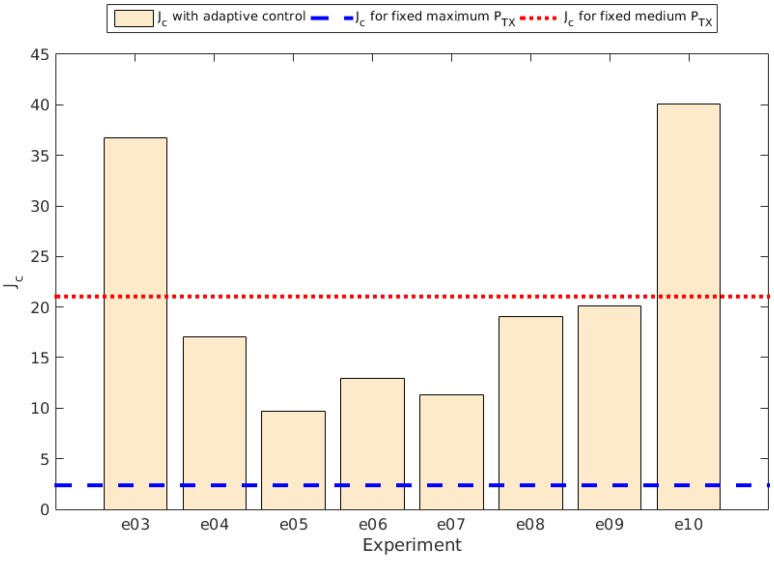
$J_{c}$ for total transmission error rate per experiment comparison.

And Figure 11 shows the comparison for the $J_{c}$ value obtained for the total transmission error rate per experiment. The values for $J_{c}$, $\alpha_{c}$ and $\beta_{c}$ per experiment are also shown in Table 4.

**Table 4:** Values of the evolution for the total error rate per experiment.

Experiment	Per Experiment			Per Round Per Experiment		
	$\alpha_{c}$	$\beta_{c}$	$J_{c}$	$\alpha_{c}$	$\beta_{c}$	$J_{c}$
e01	0.0430	0.0083	2.3734	0.0656	0.0394	1.2500
e02	0.3945	0.0034	21.0463	0.4077	0.0075	13.7053
e03	0.9533	0.0127	36.7036	0.9648	0.0293	21.6875
e04	0.5984	0.0167	17.0662	0.4996	0.0250	12.0170
e05	0.7465	0.0715	9.6857	1.3315	0.4105	4.3095
e06	0.6219	0.0354	12.9013	0.5317	0.0683	7.0283
e07	0.5859	0.0408	11.3045	0.6189	0.1179	5.5803
e08	0.8534	0.0310	19.0762	1.0577	0.0994	10.3661
e09	0.8616	0.0323	20.1254	1.1212	0.1123	9.4375
e10	0.8251	0.0704	40.1113	0.7679	0.0099	24.5000

As expected, at the beginning of each experiment there is an adjustment time that shows a dependence on the parameters used in the control system. At this point we can notice in the last experiment there is a huge error rate. There are two aspects that had its effect on this:

1. The parameters chosen for this experiment allowed the nodes to stop adjusting their transmission power when the reached 2±1 neighbors. This, in fact, caused that some of the nodes created isolated islands unable to communicate with the rest of the network.
2. Two nodes readjusted their $ND_{R}$ value at some point in the middle of the experiment, as their batteries reached values below the selected $E_{CR}$. This worsened the situation, as these two nodes were allowed to remain at a given transmission power if they reached at least one or two neighbors.

Another remarkable result is that at a fixed transmission power set to a medium one, the error rate seemed to be high and constant, so almost every experiment with the self-adaptive system reached better error rates.

**Figure 12:**
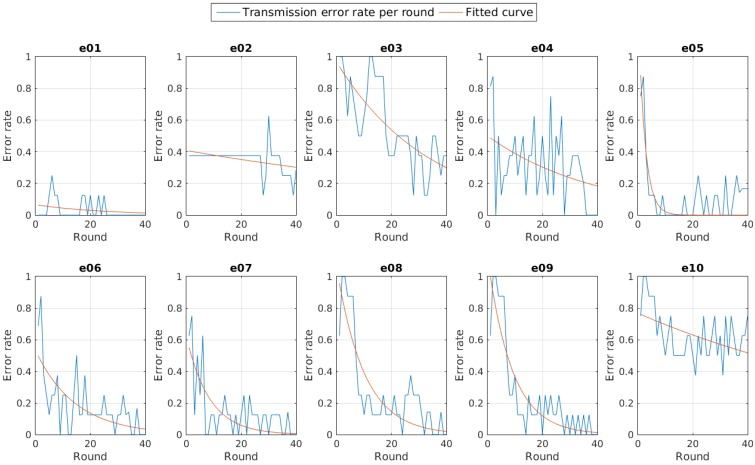
Round transmission error rate per experiment.

However, the total error rate only shows us the evolution of the network connectivity. The error is high at the beginning, given that all the nodes started from the lowest transmission power available in the adaptive experiments. This implies that although the connectivity can be good enough at a given instant, the accumulative will show just a little improvement. To realize how good the connectivity is, we explore the error rate for each iteration, that is, the current error rate per round, as shown in Figure 12.

**Figure 13:**
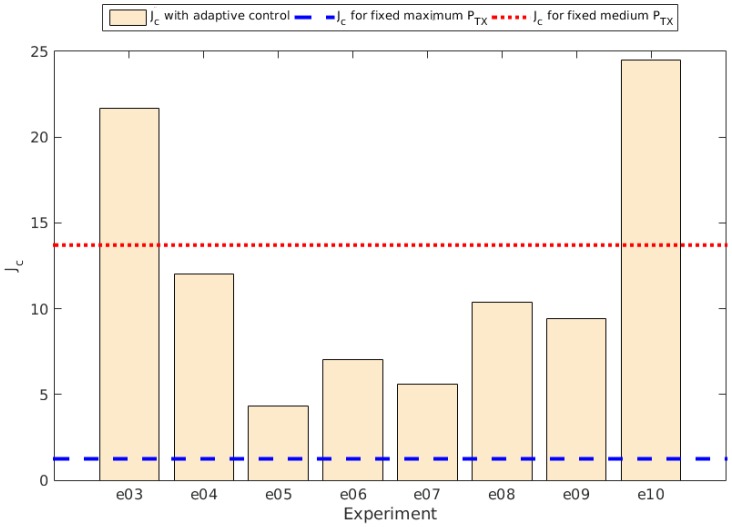
$J_{c}$ for current transmission error rate per round per experiment comparison.

Again we see that the distribution of the error rate follows a negative exponential for those experiments using the adaptive control system. Then the Equation (3) and Equation (4) are still valid for the new results. Using them we obtained the results also shown in Table 4, as well as the $J_{c}$ values comparison shown in Figure 13.

At this point we can conclude that given enough time to adjust the nodes to the new parameters, the adaptive system provides better connectivity than using a fixed transmission power fixed to a medium value, but still they have worse results than using the maximum transmission power.

## 5. Conclusions

We have observed in the results that when all nodes in a WSN are using a fixed transmission power, the system exhibits either a good connectivity or good power savings, but not both at the same time. Thereby in the first part of the results analysis we have observed that using the higher transmission power available means having the poorest power saving, but still we have nearly a perfect connectivity. On the other hand, if we choose to use a medium transmission power, we get the better power saving of all the experiments, but with a very poor connectivity. From the same observations we can conclude that experiment *e05* offers the best trade-off between energy saving and communications reliability. Also experiments *e06* and *e07* offer good results. These three experiments have in common that they use a high value for the $k_{CR}$ parameter, that is related to the variation rate of the transmission power. In future works we shall explore for the definition of a performance index for the estimation of the better trade-off of energy and communications reliability.

The fuzzy control based self-adaptive system allows the nodes in the network to achieve a balance between those two targets. It can provide a good enough power saving while keeping a high reliability of the communications. And this is specially useful when the network topology is random, and/or prone to changes, like networks with mobile nodes. Anyway the parameters with the best and the worst results in the experiments described in this paper should be tested in longer experiments.

Another open issue that worth exploring is to study how the network react to unexpected changes, like new nodes in the neighborhood, or known nodes disappearing for whatever reason. It is expected that the self-adaptive system reacts properly reaching an steady state after a while. And this is also an important issue: how long does it take to the network to reach an steady or equilibrium state?

In our experiments, we have used a fixed interval at each node for monitoring the state of the network. Of course this has influence on the observation on the current dynamic behavior of the network, and on how long it takes for the transient period. However, it can also be updated adaptively. For instance, the interval can be lower when the network is in the transient period, and higher when it has reached a steady state.

A deeper knowledge about what is the best solution will require different studies related to the dynamic behavior of networks following also cyber-physical systems approaches. The rationality of this proposal is that the dynamic behavior of the network is influenced by the topology, the physical environment, the computational load of the nodes and their processing capacity, among others.
